# Response of Extracellular Enzyme Stoichiometric Properties and Microbial Metabolic Limitations to the Ecosystem Transition Mode Employed in Red Jujube Economic Forests on the Loess Plateau

**DOI:** 10.3390/microorganisms13040729

**Published:** 2025-03-24

**Authors:** Chunheng Mu, Jiaqi Li, Fuchao Huang, Zhiyu Zhang, Jing Qin, Gailing Wang

**Affiliations:** 1 College of Resources and Environment, Shanxi Agricultural University, Taigu 030801, China; muchunheng@163.com (C.M.); ljq2370208799@163.com (J.L.); 17839803157@163.com (F.H.); 13203516841@163.com (J.Q.); 2 State Key Laboratory of Black Soils Conservation and Utilization, Northeast Institute of Geography and Agroecology, Chinese Academy of Sciences, Changchun 130012, China; zhangzhiyu@iga.ac.cn

**Keywords:** soil enzyme activity, vector model, microbial nutrient limitation, red date forest

## Abstract

Soil carbon (C), nitrogen (N), and phosphorus (P) cycling and microbial metabolism limitations are key factors affecting nutrient cycling and vegetation development. Extracellular enzyme activity (EEA) plays a key role in carbon and nutrient cycling in ecosystems, and their activities can serve as indicators of microbial nutrient requirements. At present, there is insufficient research on the nutrient limitations of microorganisms during ecosystem transition in abandoned jujube forests on the Loess Plateau. Four modes were selected: jujube forest replanted with Pinus tabulaeformis (CP), with Platycladus orientalis (PO), with medicinal materials (MM), and with alfalfa (AL). An abandoned jujube forest (CK) was used as a control. Soil physical and chemical properties, microbial biomass carbon, nitrogen, and phosphorus, as well as changes in the activities of β-1,4-glucosidase (BG), leucine aminopeptidase (LAP), N-acetylglucosamine (NAG), and alkaline phosphatase (AP), were studied. Analysis of changes in soil microbial nutrient limitations was performed. Compared with those in the CK treatment, the activities of soil C, N, and P extracellular enzymes significantly increased (*p* < 0.05) in the forest transition treatments, and the C:N_EEA_, C:P_EEA_, and N:P_EEA_ ratios of extracellular enzymes tended to decrease. Within the treatments, the activities of soil C, N, and P extracellular enzymes decreased as the soil layer deepened, whereas the enzyme stoichiometric ratio increased as the soil layer deepened, with significant differences observed between the soil layers. The vector model was used to quantify nutrient limitations in microbial metabolism and revealed that microbial metabolism in surface soil was limited mainly by C and P and that in the 10–20 cm and 20–40 cm layers, soil microbial metabolism was limited mainly by C and N. Correlation analysis revealed that SOC, pH, MBC, and MBN were the main factors affecting soil extracellular enzyme activity. Mantel’s test revealed that (NAG + LAP), AP, C:N_EEA_, and C:P_EEA_ were important factors affecting vector length and angle. RAD analysis revealed that microbial properties had a greater impact on enzyme stoichiometry and microbial metabolic limitations than physicochemical indicators did. This study highlights the importance of vegetation in determining microbial metabolic processes and enhances our understanding of how ecological changes in jujube forests affect soil nutrient cycling and microbial metabolic constraints on the Loess Plateau. Forest transformation modes have important impacts on soil extracellular enzyme activity and microbial nutrient limitation.

## 1. Introduction

Soil microorganisms play crucial roles in terrestrial ecosystems by dominating soil life activities, which include the decomposition of organic matter, the regulation of nutrient cycles, and the enhancement of soil structure. These processes collectively fulfill a range of essential ecological functions within soil [1,2,3]. Microorganisms have mechanisms for maintaining elemental homeostasis [4]. When an imbalance occurs between microbial metabolic requirements and nutrient availability in soil, microbial metabolism can be constrained by soil nutrients. This limitation not only impacts the metabolic rate of microorganisms but also influences the allocation patterns of resources within their metabolic processes [5,6]. Microbial metabolic nutrient limitation is an important driving force for carbon (C) and nutrient cycling in terrestrial ecosystems [7] and is a key factor affecting biogeochemical cycles. Soil extracellular enzyme activity (EEA) originates primarily from microbial activities and root secretions [8]. EEAs catalyze the decomposition and transformation of soil organic matter, facilitating the release of inorganic nutrients that can be absorbed and utilized by plants and microorganisms. This process provides a continuous supply of energy and nutrients for the soil ecosystem. Studying soil extracellular enzyme activity can elucidate the balance and interaction between microbial nutrient limitations and soil nutrients, thereby providing a scientific basis for the evaluation and management of soil quality.

In recent years, the application of chemometrics in soil enzyme research has become increasingly widespread, offering robust support for in-depth investigations into the functions of soil enzymes and their relationship with soil nutrient cycling. Numerous enzymes in soil participate in the depolymerization and catabolism of complex polymers, such as cellulose; however, only a limited number of these enzymes ultimately catalyze terminal hydrolysis reactions [4]. β-1,4-glucosidase (BG) promotes the degradation of cellulose, whereas β-1,4-N-acetylglucosaminidase (NAG) and l-leucine aminopeptidase (LAP) facilitate the degradation of chitin and protein, respectively. Alkaline phosphatase (AP) is involved in the degradation of organophosphorus compounds [4,5,9]. These extracellular enzymes are commonly utilized to represent the activities of carbon-acquiring, nitrogen-acquiring, and phosphorus-acquiring enzymes. Soil extracellular enzymes (EES) stoichiometry serves as a sensitive indicator of microbial metabolic responses to limitations in energy and nutrients [10,11]. The constraints of carbon (C), nitrogen (N), and phosphorus (P) in soil can be elucidated by analyzing the ratios of the activities of enzymes involved in nutrient acquisition [7,12,13]. Globally, the average stoichiometric ratio of microbial metabolic carbon (C), nitrogen (N), and phosphorus (P) in extracellular enzymes within terrestrial soils is approximately 1:1:1 [4]. This ratio is considered the equilibrium point for soil extracellular enzyme activity [4,14]. However, since soil enzyme activity is susceptible to environmental factors, such as vegetation type, organic carbon content, pH, microbial biomass [15,16,17], and soil depth [18,19], the C:N:P ratio deviates from 1:1:1 [9,20,21], thereby affecting microbial metabolism.

Moorhead et al. [12,14] constructed a soil enzyme stoichiometry vector model. The ordinate y represents the ratio of C-acquiring enzyme activity relative to N-acquiring enzyme activity, and the abscissa x represents the ratio of C-acquiring enzyme activity relative to P-acquiring enzyme activity. The lines connecting the point (x, y) with the origin (0, 0) are vectors [14,16]. C limitation in soil is represented by vector length, and N and P limitation in soil is represented by vector angle [12]. The vector lengths and vector angles in enzyme stoichiometry provide new way to determine nutrient limitations in microbial metabolism. During the secondary succession of grasslands on the Loess Plateau, soil microorganisms are limited by both N and P, with a shift from P limitation to N limitation as succession continues [22]. Li et al. [23] used vector models to reveal that microbial metabolism is limited mainly by N and P in abandoned terraced fields in southern China. During the secondary succession process of northern forests in China, vegetation type has a significant effect on soil enzyme activity and stoichiometry [24]. Some scholars have also reported large differences in the effects of physical and chemical factors and biological factors on enzyme activity and microbial metabolism limitations [6,25]. Compared with plant characteristics and soil characteristics, soil microbial factors are better able to explain changes in soil ecological enzyme activity and stoichiometry [21]. Xu et al. [5] found that climatic factors have the greatest impact on soil microbial restriction in the desert areas of western China, followed by soil abiotic factors and finally by biotic factors.

The Loess Plateau of China is one of the most fragile environments in the world, with low soil nutrient availability and severe soil erosion [25,26,27,28]. It is also an important part of China’s “Three Zones and Four Belts” ecological security strategy. The Lüliang Mountains are the main component of the Loess Plateau. As an important ecological project and traditional industry for water and soil erosion control in loess hilly areas, red date (Ziziphus jujuba) economic forests have a planting area of approximately 13.33 × 10^4^ hm^2^ in Shanxi Province, 40% of which is in the Lüliang Mountains. However, with societal development and increases in labor costs, the economic benefits of red dates have declined significantly; as a result, most of the red date economic forests in the Lüliang Mountains have been abandoned. In the Returning Farmland to Forest initiative implemented in 2020, Lüliang city included degraded red date economic forests within the scope of farmlands to be returned to forests; that is, other species were planted in the red date economic forests, and the economic forests were transitioned to ecological forests. Some scholars have studied the enzyme stoichiometry of surface soils on the Loess Plateau; however, there have been no reports on the differences in soil extracellular enzyme stoichiometry, microbial nutrient limitations, or their driving factors under different ecosystem transition models in the Lüliang Mountains. Studying soil extracellular enzyme activity and enzyme stoichiometry characteristics under different ecosystem transition modes in jujube forests can help determine microbial nutrient limitations and soil nutrient cycling properties. The main objectives of this study are: (1) to study the changes in the activities of soil C, N, and P extracellular enzymes and their stoichiometric characteristics under different vegetation types during ecosystem transition in the red jujube economic forests in the Lüliang Mountains; (2) to clarify soil C and nutrient limitation status under different ecosystem transition modes on the basis of soil enzyme stoichiometry and vector characteristics; and (3) to determine the key factors affecting soil microbial metabolism under different ecosystem transition modes.

## 2. Materials and Methods

### 2.1. Study Area Information

The study area was located in Linxian County, Lüliang city, Shanxi Province, on the central Loess Plateau of China (37°35′52″–38°14′19″ N, 110°39′40″–111°18′02″ E). It has a temperate semiarid climate, with an average annual precipitation of 519 mm (which mainly occurs from July to September), high annual evaporation, the rate of which is four times greater than that of precipitation, an average annual sunshine duration of 2807 h, and a frost-free period of 160 days [29]. The soil type is mainly loess (calciferous loess, FAO classification) [30], which is developed from aeolian loess parent material. It has yellow particles, no layering, and a silty texture, is loose and barren, has poor resistance to erosion, and shows severe erosion. This area is the largest red date production base in Shanxi Province and is known as the “Hometown of Chinese Red Dates”. In recent years, owing to the increase in input costs and low income, some jujube forests have been abandoned.

### 2.2. Experimental Design and Soil Sampling

Sampling was carried out in October 2023. Through a review of relevant information and in-depth interviews with local villagers on site, plots with five types of jujube forest ecosystem transition models with similar slopes, aspects, and altitudes were selected, namely, red jujube forest replanted with pine (CP), with Platycladus arborvitae (PO), with alfalfa (AL), with medicinal materials (MM), and abandoned jujube forest (CK). In these plots, the age of the jujube forests was greater than 20 years, and the age of various vegetation planted and the age of the abandoned plot was 6 years. The sample area was 2000 m^2^. Before ecosystem transition, the density of the jujube forest was 600 plants·hm^−2^, the spacing between plants was 3 m, and the spacing between rows was 5 m. The jujube forests replanted with CP and PO were replanted with different plants in the rows. The ratio of jujube forest to pine and arborvitae was 1:3. The medicinal materials Astragalus-Bupleurum (MM) and AL were planted in the jujube forests using hole-sowing, and the sowing density was 9 holes·m^−2^. The abandoned jujube forests (CK) did not require any management measures, and weeds grew freely. Before ecosystem transition, the jujube forest was not removed or altered. After the transition, there was no fertilization or human intervention, and the plants were allowed to grow naturally. The dominant grass species included black nightshade, goosegrass, Artemisia sacrorum, and Aster hispidus. Three quadrats (20 × 20 m) were randomly set up in each ecosystem transition plot. After the surface litter layer and other debris were removed, a soil auger with a diameter of 5 cm was used to collect soil samples from the 0–10 cm, 10–20 cm, and 20–40 cm layers in each quadrat using an S-shaped five-point sampling method. The soil samples were divided into two parts: one part was air-dried for physical and chemical property and enzyme activity determination, and the remaining part was immediately stored in a refrigerator at 4 °C for microbial biomass determination.

### 2.3. Soil Characterization

The soil organic carbon (SOC) content was analyzed using the potassium dichromate oxidation external heating method. The soil total nitrogen (TN) content was determined by the Kjeldahl method [31]. The total phosphorus (TP) content was determined using the perchloric acid–sulfuric acid method. Available nitrogen (AN) was measured using the alkaline hydrolysis diffusion method [32]. The available phosphorus (AP) content was determined via the sodium bicarbonate leaching–molybdenum antimony colorimetric method. Soil pH was measured using a pH meter (Lower Saxony, Germany), and the ratio of soil to water was 1:2.5.

Soil microbial biomass carbon (MBC) and microbial biomass nitrogen (MBN) were measured using chloroform fumigation–K_2_SO_4_ extraction and a multi N/C 3100 TOC meter (Analytik, Jena, Germany). The soil microbial biomass phosphorus (MBP) content was determined using chloroform fumigation–molybdenum antimony colorimetry [33].

A kit was used to measure the activities of the soil enzymes β-1,4-glucosidase (BG), β-1,4-N-acetylglucosaminidase (NAG), leucine aminopeptidase (LAP), and alkaline phosphatase (AP). Enzyme activity is expressed in μmol·h^−1^·g^−1^ sample. The kit was produced by Shanghai Enzyme Biotechnology Co., Ltd. (Shanghai, China), and the analysis procedure was carried out in accordance with the manufacturer’s instructions. The C acquisition enzyme activity is expressed as BG, the N acquisition enzyme activity is expressed as NAG + LAP, and the P acquisition enzyme activity is expressed as AP.

### 2.4. Calculation of Microbial Nutrient Limitations

The activities of soil C, N, and P enzymes were measured to obtain the stoichiometric ratios of the enzymes. The formulas are as follows:

C:N_EEA_ = ln(BG):ln(NAG + LAP) (1)

C:P_EEA_ = ln(BG):ln(AP) (2)

N:P_EEA_ = ln(NAG + LAP):ln(AP) (3)

A vector model was constructed to illustrate the nutrient limitation status of microorganisms. The vector length and vector angle were generated by plotting C:N and C:P to obtain the enzyme ratio diagram [12]. A longer vector length means that microorganisms are more limited by C. When the vector angle > 45°, it represents relative P limitation, and when the vector angle < 45°, it represents relative N limitation. In this study, microbial metabolic limitations were quantified for all data by constructing a vector model using natural logarithmic transformation of the extracellular enzyme activity ratios [15,34]. The vector length and angle were calculated by Formulas (4) and (5), respectively:

Vector length = SQRT{(lnBG/lnAP)^2^ + [lnBG/ln(NAG + LAP)]^2^} (4)

Vector angle = Degrees {ATAN2 [lnBG/lnAP, lnBG/ln(NAG + LAP)]}(5)

Ecological stoichiometry theory shows that although the proportions of C, N, and P in soil vary greatly, the C:N:P ratio in microbial biomass is strictly limited, which is the so-called stoichiometric steady state of the microbial community. The formula is as follows:

H′ = 1/m

where m is the regression slope between ln (SOC:TN) and ln (MBC:MBN) or the slope between ln (SOC:TP) and ln (MBC:MBP). The slope can be used to represent the degree of microbial elemental stability, where H′ ≫ 1 represents strong stoichiometric stability and H′ ≈ 1 represents weak stability [24].

### 2.5. Data Analysis

The one-way analysis of variance (ANOVA) module in SPSS 26.0 (version 26.0; SPSS Inc., Chicago, IL, USA) was used to test the differences in each index under the different treatments. Redundancy analysis (RDA) was used to study the main factors affecting soil enzyme stoichiometric ratios and vector characteristics. Prior to RDA, Canoco 5 (Canoco, NY, USA) software was used to rank the environmental factors and screen for factors with high explanatory value for determining enzyme stoichiometry. Origin 2021 (Origin Lab Corporation, Northampton, MA, USA) was used to conduct linear regression analysis and histogram plotting of enzyme activity. The gene cloud tools platform was used to perform Mantel tests and Spearman’s analyses between vector length and angle and soil physical and chemical properties and microbial characteristics.

## 3. Results

### 3.1. Soil C, N, P, Microbial Biomass C, N, P, and Their Measurement Ratios in Different Ecosystem Transitions

**Figure 1:**
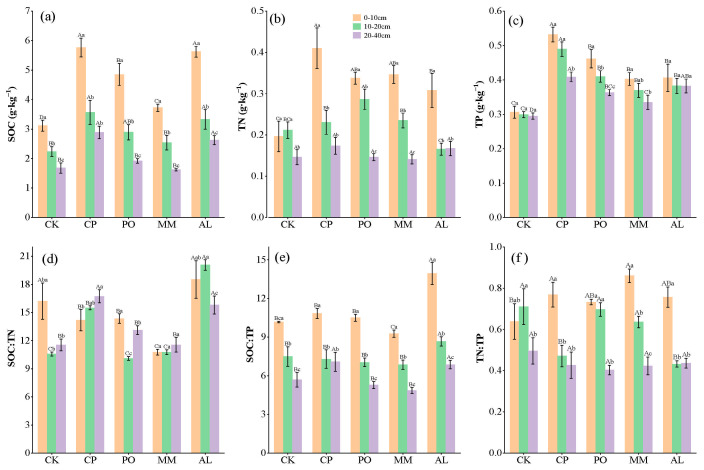
Soil C, N, and P contents and their stoichiometric ratios. (**a**) SOC, soil organic carbon; (**b**) TN, total nitrogen; (**c**) TP, total phosphorus; (**d**) SOC:TN, ratio of SOC to TN; (**e**) SOC:TP, ratio of SOC to TP; (**f**) TN:TP, ratio of TN to TP. Different lowercase letters indicate significant differences between different soil layers of the same treatment (*p* < 0.05) and different uppercase letters indicate significant differences between different treatments of the same soil layer (*p* < 0.05).

Compared with those in the CK treatment, the soil organic carbon, total nitrogen (TN), and total phosphorus (TP) contents in the CP, PO, MM, and AL treatments increased after ecosystem transition, among which the organic carbon contents in the CP and AL treatments increased significantly. The CP treatment had the highest TN and TP contents, and the SOC, TN, and TP contents all decreased with increasing soil depth. The SOC:TN and SOC:TP ratios were the highest in AL, significantly higher than those in the other four plots, and the SOC:TP and TN:TP ratios decreased as the soil depth increased (Figure 1).

**Figure 2:**
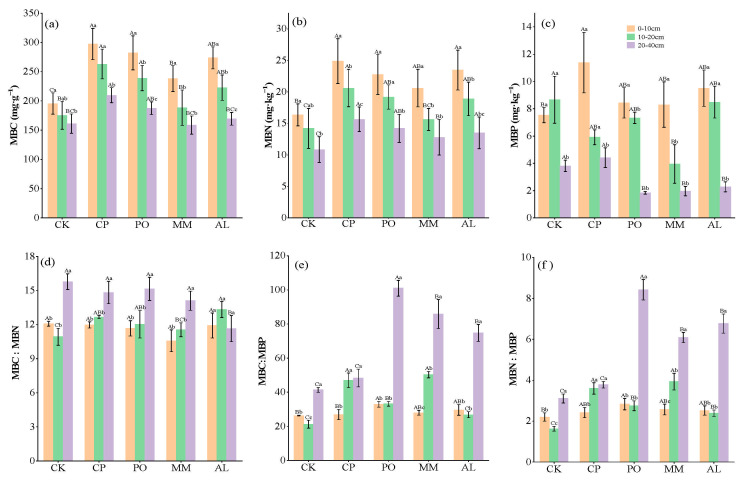
Soil microbial biomass C, N, and P content and their stoichiometric ratios. (**a**) MBC, microbial biomass carbon; (**b**) MBN, microbial biomass nitrogen; (**c**) MBP, microbial biomass phosphorus; (**d**) MBC:MBN, ratio of MBC to MBN; (**e**) MBC:MBP, ratio of MBC to MBP; (**f**) MBN:MBP, ratio of MBN to MBP. Different lowercase letters indicate significant differences between different soil layers of the same treatment (*p* < 0.05) and different uppercase letters indicate significant differences between different treatments of the same soil layer (*p* < 0.05).

The contents of soil MBC, MBN, and MBP were affected by transition mode and soil depth. The contents of MBC and MBN among the various soil layers were highest in the CP treatment and lowest in the CK treatment, and there were significant differences among the various plots and soil layers (*p* < 0.05, Figure 2a–c). In terms of the stoichiometric ratio, except for that in the AL treatment, the MBC:MBN ratio increased with increasing depth of the soil layer and was not significantly different among treatments for the same soil layer. MBC:MBP was the lowest in the CK treatment in the three soil layers and increased with increasing depth of the soil layer. There were significant differences in MBC:MBP between the various plots (*p* < 0.05). The MM treatment resulted in the highest MBC:MBP ratio in the 10–20 cm soil layer (Figure 2d–f).

### 3.2. Changes in Soil Extracellular Enzyme Activity and Stoichiometry Under Different Ecosystem Transition Models

**Figure 3:**
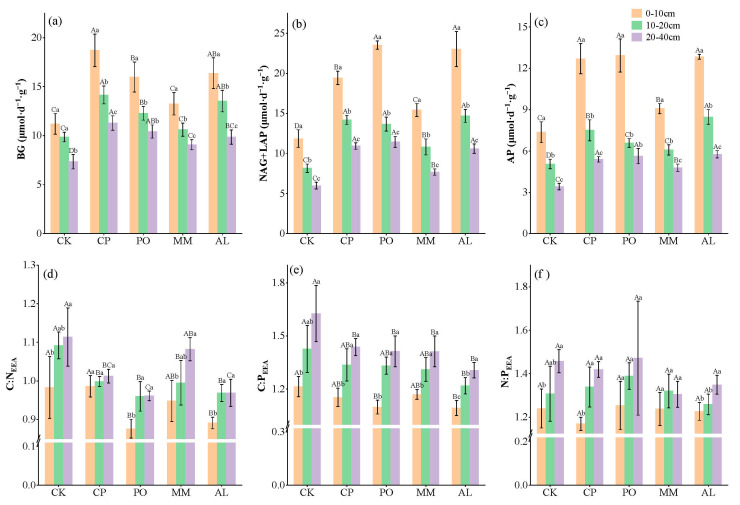
Soil C, N, P acquisition enzyme activities and stoichiometric ratios. (**a**) BG, C acquisition enzyme; (**b**) NAG + LAP, N acquisition enzyme; (**c**) AP, P acquisition enzyme; (**d**) C:N_EEA_, ratio of log-transformed activity of BG to that of NAG + LAP; (**e**) C:P_EEA_, ratio of log-transformed activity of BG to that of AP; (**f**) N:P_EEA_, ratio of log-transformed activity of NAG + LAP to that of AP. Different lowercase letters indicate significant differences between different soil layers of the same treatment (*p* < 0.05) and different uppercase letters indicate significant differences between different treatments of the same soil layer (*p* < 0.05).

Compared with those in the CK treatment, the extracellular enzyme activities of C, N, and P increased overall. In the 0–10 cm soil layer, the soil BG enzyme activity in the CP treatment was 18.72 μmol·h^−1^·g^−1^, that in the AL treatment was 16.38 μmol·h^−1^·g^−1^, and that in the PO treatment was 16.00 μmol·h^−1^·g^−1^, all of which were significantly greater than the enzyme activity in the CK treatment. Soil NAG + ALP enzyme activity in the CP treatment was 19.44 μmol·h^−1^·g^−1^, that in PO was 23.54 μmol·h^−1^·g^−1^, that in AL was 23.05 μmol·h^−1^·g^−1^, and that in MM was 15.43 μmol·h^−1^·g^−1^, which were significantly higher than that of 11.84 μmol·h^−1^·g^−1^ in CK. The soil AP activity was also significantly greater in the replanted plots than in the CK plot. The differences in C, N, and P extracellular enzyme activities in the 10–20 cm and 20–40 cm soil layers were essentially consistent with the changes in the surface layer. The enzyme activities in the various plots decreased with increasing depth of the soil layer, and there were differences in enzyme activity among soil layers (*p* < 0.05, Figure 3a–c).

In terms of extracellular enzyme stoichiometry, the average values of soil C:N_EEA_, C:P_EEA_, and N:P_EEA_ in the 0–10 cm soil layer were 0.87~0.99, 1.09~1.21, and 1.17~1.26, respectively. Compared with those in the CK treatment, the C:N_EEA_ and C:P_EEA_ ratios in the PO and AL treatments and the C:P_EEA_ ratio in the AL treatment were significantly lower (*p* < 0.05, Figure 3e,f). There was no significant difference between C:P_EEA_ and N:P_EEA_ in the CP, PO, AL, and MM treatments; however, the C:N_EEA_ of CP was significantly greater than that of PO and AL (*p* < 0.05, Figure 3d–f). In contrast to enzyme activity, the values of the enzyme stoichiometric ratios increased with increasing depth of the soil layer. In the other plots, the C:N_EEA_ and C:P_EEA_ ratios were significantly lower than those in the CK in the 10–20 cm and 20–40 cm soil layers, and the N:P_EEA_ ratio was not significantly different among the various plots (Figure 3d–f).

### 3.3. Soil Microbial Nutrient Limitations and Homeostasis Under Different Ecosystem Transition Models

**Figure 4:**
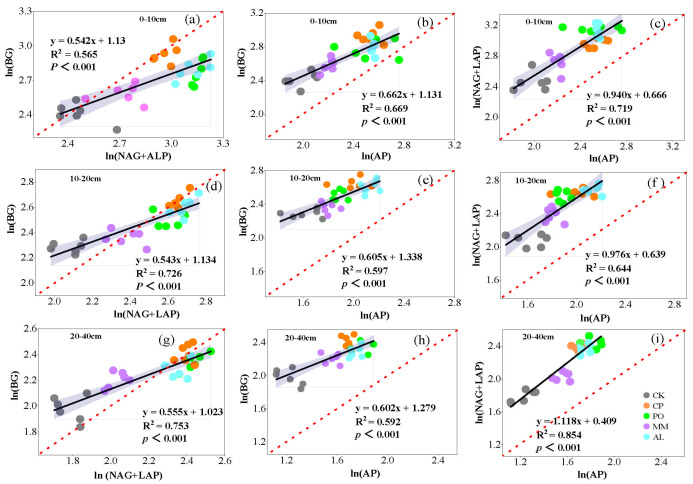
Linear regression analysis between soil extracellular enzyme stoichiometry of C, N, and P. (**a**,**d**,**g**) ratio of log-transformed activity of NAG + LAP to BG for 0–10 cm, 10–20 cm, and 20–40 cm, respectively; (**b**,**e**,**h**) ratio of log-transformed activity of AP to BG for 0–10 cm, 10–20 cm, and 20–40 cm, respectively; (**c**,**f**,**i**) ratio of log-transformed activity of AP to NAG + LAP for 0–10 cm, 10–20 cm, and 20–40 cm, respectively.

Soil microbial nutrient limitation can be characterized by the stoichiometric ratio of extracellular enzymes, that is, the ratio of the intercept sizes of ln(BG) to ln(NAG + LAP), ln(BG) to ln(AP), and ln(NAG + LAP) to ln(AP). The three intercepts in the regression analysis represent the initial C limitation (relative to N), the initial P limitation (relative to C), and the initial N limitation (relative to P), respectively. In the 0–10 cm soil layer, the intercepts of the three regression lines of ln(BG) and ln(NAG + LAP), ln(BG) and ln(AP), and ln(NAG + LAP) and ln(AP) were I_C:N_(1.130), I_C:P_(1.131), and I_N:P_(0.666); in the 10–20 cm soil layer, the intercepts were I_C:N_(1.134), I_C:P_(1.338), and I_N:P_(0.639); and the intercepts in the 20–40 cm soil layer were I_C:N_(1.023), I_C:P_(1.279), and I_N:P_(0.409). Regression analysis of soil C, N, and P extracellular enzymes under different ecosystem transition models revealed that there was a strong positive correlation between enzymes (Figure 3a–c, *p* < 0.001). In addition, the slopes of ln(BG) and ln(NAG + LAP) from 0–10 cm were 0.542, those from 10–20 cm were 0.543, and those from 20–40 cm were 0.555; the slopes of ln(BG) and ln(AP) from 0–10 cm were 0.662, those from 10–20 cm were 0.605, and those from 20–40 cm were 0.602. The slopes of the three soil layers all deviated from the 1:1 ratio of C:N_EEA_ and C:P_EEA_ at the global scale [7] (Figure 4).

**Figure 5:**
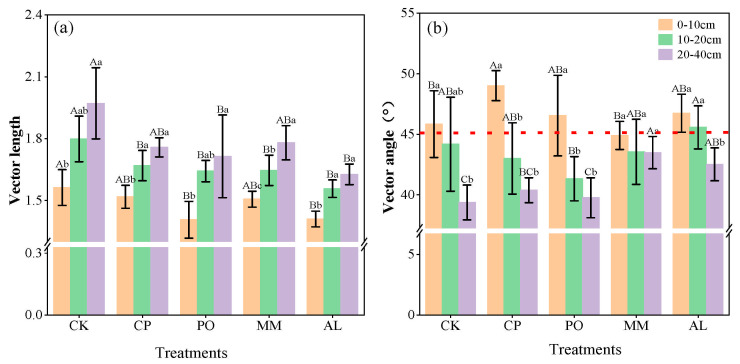
Vector lengths and angles. (**a**) vector length; (**b**) vector angle. Different lowercase letters indicate significant differences between different soil layers of the same treatment (*p* < 0.05) and different uppercase letters indicate significant differences between different treatments of the same soil layer (*p* < 0.05).

**Figure 6:**
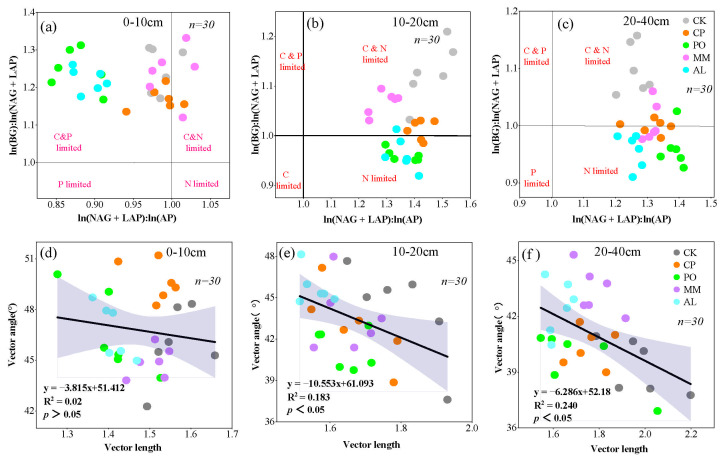
Scatterplot of soil enzyme stoichiometry limits and the relationship between vector length and vector angle. (**a**–**c**) Scatter plots of enzyme stoichiometric limits for 0–10 cm, 10–20 cm, and 20–40 cm, respectively; (**d**–**f**) relationship between vector length and vector angle for 0–10 cm, 10–20 cm and 20–40 cm, respectively. The figure *n* = 30 refers to a total of 5 treatments in each subplot, with 6 replicates per treatment, for a total of 30 data.

The vector model is a method used to characterize microbial nutrient limitations [15]. According to the vector model, the vector length of the CK treatment was significantly greater than those of the other treatments across the different plots and soil layers. There were no significant differences among the CP, PO, MM, and AL treatments (Figure 5a), indicating that CK was most limited by C. As the soil layer deepened in various fields, C limitations also intensified. With the exception of the MM treatment, in which the vector angle was less than <45° and the soil was limited by N, the vector angles of the treatments in the 0–10 cm soil layer were all greater than 45°, showing P limitation of soil microbial metabolism. Among them, the CP treatment had a value of 49.7°, which was the most limited. This finding is consistent with the scatterplot of enzyme stoichiometric values, which shows that most soil samples fell within the C and P co-limited quadrant (Figure 6a). Except for the AL treatment, all plots in the 10–20 cm soil layer were limited by N. All plots in the 20–40 cm soil layer were limited by N, and there were significant differences among the plots. As the soil layer deepened, microbial nutrient limitation shifted from P limitation to N limitation (Figure 5b and Figure 6a–c). In addition, there was no correlation between the vector length and the vector angle in the 0–10 cm soil layer (Figure 6d), but there was a significant negative correlation between the vector length and the vector angle in the 10–20 cm and 20–40 cm soil layers (*p* < 0.05, Figure 6e,f).

**Figure 7:**
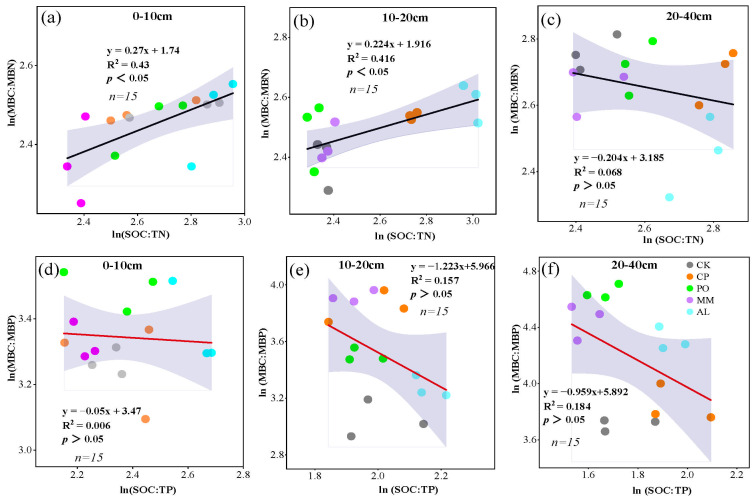
Soil microbial community homeostasis. (**a**–**c**) Steady state between ln(SOC:TN) and ln(MBC:MBN) for 0–10 cm, 10–20 cm, and 20–40 cm, respectively; (**d**–**f**) steady state between ln(SOC:TP) and ln(MBC:MBP) for 0–10 cm, 10–20 cm, and 20–40 cm, respectively. The figure *n* = 15 refers to a total of 5 treatments with 3 replications per treatment for a total of 15 data in each subplot.

By analyzing the correlations between the microbial biomass C, N, and P ratios and the soil C, N, and P ratios, the dynamic stoichiometric balance at the community level was examined. The slopes of ln(MBC:MBN) and ln(SOC:TN) were 0.270 at 0–10 cm, 0.224 at 10–20 cm, and 0.204 at 20–40 cm (Figure 7a–c). The slopes of ln(MBC:MBP) and ln(SOC:TP) were 0.050 at 0–10 cm, 1.223 at 10–20 cm, and 0.959 at 20–40 cm (Figure 7d–f). The steady-state model (H′ = 1/m) was used to describe the stoichiometric steady state. The H′ values of ln(MBC:MBN) and ln(SOCC:TN) were 3.7 at 0–10 cm and 4.5 at 10–20 cm, showing weak stability and significant correlations (*p* < 0.05). H’ was 4.9 at 20–40cm, with weak stability and no significance. The H′ of ln(MBC:MBP) and ln(SOC:TP) was 20 in 0–10cm, which showed a strong stoichiometric steady state, but there was no significant correlation. In the 10–20 cm soil layer, H′ was 0.82, and in the 20–40 cm soil layer, H′ was 1.04, which showed weak stability but had no significant correlation (Figure 7).

### 3.4. Relationships Between Soil Chemical Properties, Extracellular Enzyme Activity, and Microbial Nutrient Limitation in Different Ecosystem Transition Models

**Figure 8:**
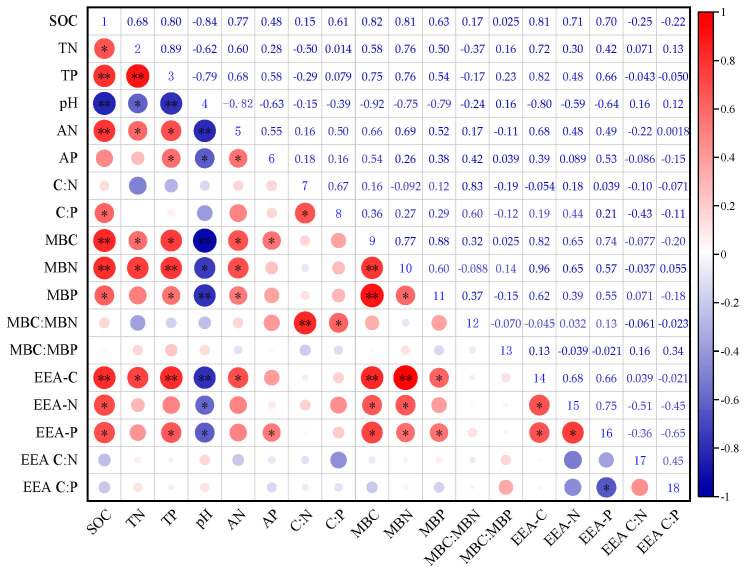
Pearson correlation analysis of soil properties and enzyme activities and stoichiometric ratios. SOC, soil organic carbon; TN, total nitrogen; TP, total phosphorus; AN, alkaline hydrolyzed nitrogen; AP, available phosphorus; C:N, soil carbon to nitrogen ratio; C:P, soil carbon to phosphorus ratio; MBC, microbial biomass carbon; MBN, microbial biomass nitrogen; MBP, microbial biomass phosphorus; MBC:MBN, ratio of MBC to MBN; MBC:MBP, ratio of MBC to MBP; MBN:MBP, ratio of MBN to MBP; EEA-C: carbon acquisition enzyme, EEA-N: nitrogen acquisition enzyme, EEA-P: phosphorus acquisition enzyme, EEA C:N: enzyme carbon to nitrogen ratio, EEA C:P: enzyme carbon to phosphorus ratio (* *p* < 0.05, ** *p* < 0.01).

**Figure 9:**
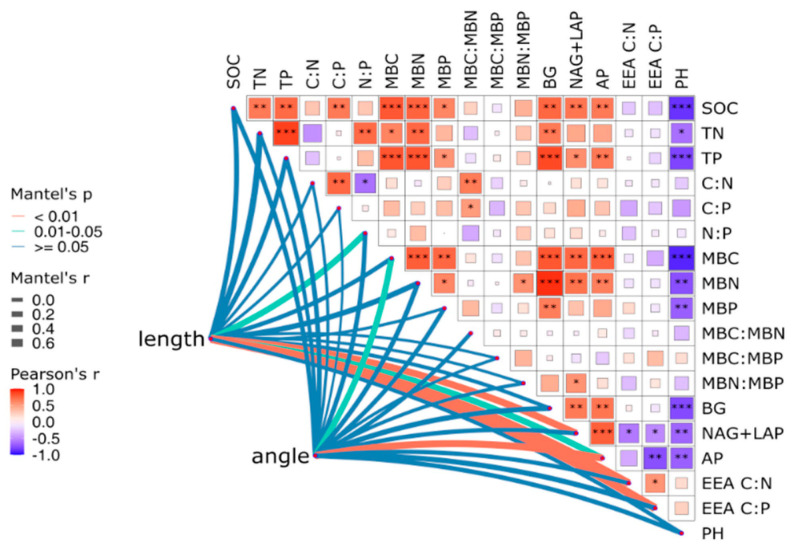
Spearman correlation between vector length and angle and soil chemical properties and microbial properties. The thickness of the line indicates the strength of the correlation, and the asterisk indicates statistical significance (* *p* < 0.05, ** *p* < 0.01, *** *p* < 0.001). Length: vector length, angle: vector angle.

Correlation analysis (Figure 8) revealed that under different ecosystem transition modes, soil BG enzyme activity had a very significant positive correlation with SOC, TP, MBC, and MBN (*p* < 0.01) and a very significant negative correlation with pH. In addition, BG enzyme activity was significantly positively correlated with TN, AN, and MNP (*p* < 0.01). The enzymes NAG + LAP and AP were significantly positively correlated with SOC, MBC, and MBN (*p* < 0.05) and significantly negatively correlated with pH. There was a significant negative correlation between the soil enzyme C:P_EEA_ ratio and AP activity (*p* < 0.05). Spearman’s correlation analysis and the Mantel test heatmap results revealed that NAG + LAP, AP, C:N_EEA_, C:P_EEA_, and soil N:P significantly changed the vector length, and AP and MBC significantly changed the vector angle (Figure 9).

**Figure 10:**
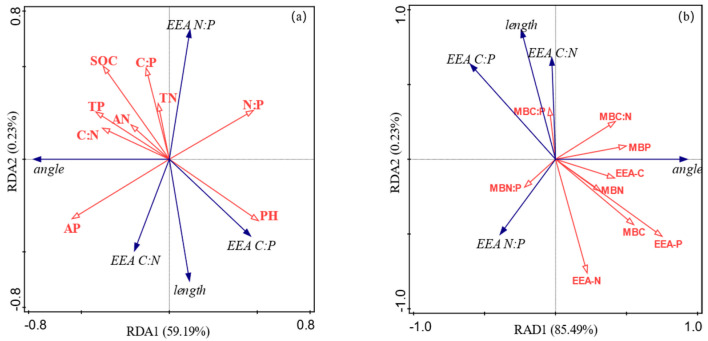
RDA analysis of soil chemical properties and microbial properties on enzyme stoichiometry and microbial nutrient limitations. (**a**) Relationship of chemical properties to enzyme stoichiometric ratios and microbial nutrient limitation; (**b**) relationship of microbial properties to enzyme stoichiometric ratios and microbial nutrient limitation.

Vector length and vector angle were selected as response variables, and soil chemical properties and microbial properties were used as explanatory variables to perform redundancy analysis. The results revealed that soil chemical properties explained 59.42% of the total variation related to extracellular enzyme stoichiometry and microbial metabolism limitations. Among them, axis 1 and axis 2 accounted for 59.19% and 0.23%, respectively (Figure 10a). Soil pH had a strong positive effect on the vector length and C:P_EEA_ ratio but a strong negative effect on the vector angle and N:P_EEA_ ratio. Soil microbial properties explained 85.72% of the total variation, for which axis 1 and axis 2 accounted for 85.49% and 0.23%, respectively (Figure 10b). The soil microbial biomass carbon, nitrogen, and phosphorus contents and the MBC:MBN, BG, and AP enzyme activities had strong positive impacts on the vector angle but had strong negative effects on the C:P_EEA_ ratio, and the vector length was affected mainly by the MBC:MBP ratio. These findings indicate that enzyme stoichiometry and microbial metabolism limitations are more affected by microbial properties than by physical and chemical properties.

## 4. Discussion

### 4.1. Effects of Different Ecosystem Transition Models on Soil Extracellular Enzyme Activities

Studies have shown that, compared with CK, revegetation with tree species significantly increased soil C acquisition enzyme (BG), N acquisition enzyme (NAG + LAP), and P acquisition enzyme (AP) activities. Vegetation type can lead to changes in soil microbial biomass and soil physical and chemical properties (such as soil organic carbon, soil available phosphorus and moisture, etc.) [15,24,35]. These changes can directly or indirectly affect the production of microbial enzymes, leading to different changes in enzyme activity under different vegetation levels [36,37]. The activities of C, N, and P acquisition enzymes in the CP and PO treatments were significantly greater than those in the MM treatment, possibly because trees have more developed root systems than herbaceous plants do. Trees release more exudates, which results in higher soil acquisition enzyme activity [38]. Studies have shown that vegetation with greater root biomass has higher activities of C-, N-, and P-acquiring enzymes [39]. In addition, mixed, high-quality litter easily decomposes and provides more nutrients than litter from individual tree species does, providing abundant substrates for microorganisms to produce enzymes and helping the microbial community transform from an oligotrophic community to a eutrophic community [21,40]. The CK treatment resulted in less vegetation and less developed root systems, which is why the enzyme activity was lower than that in the other plots.

Notably, although alfalfa, an herbaceous plant, was planted in the AL plot, the activity of N acquisition enzymes (NAG + LAP) was high in this plot. This may have occurred because alfalfa is a leguminous plant with abundant root hairs and large root biomass, especially fine roots [41]. It has strong nitrogen-fixing capacity, provides sufficient N for alfalfa, and promotes the growth and activity of microorganisms [21,41]. Therefore, vegetation type has an important influence on soil enzyme activity.

In the 0–10 cm, 10–20 cm, and 20–40 cm soil layers, the activities of all extracellular enzymes decreased with increasing depth. The main reason was that the replanted vegetation was young, the root systems of the vegetation were still expanding, and the litter was distributed mainly in the surface soil. When plants provide large amounts of root exudates, microorganisms decompose litter to form humus and release nutrients [42]. These nutrients first accumulate at the soil surface, which results in a relatively high nutrient content at the soil surface. High soil nutrient content promotes microbial activity and growth [17]. As the soil layer deepens, the organic matter content and nutrient utilization rate in the soil decrease [43], and the enzyme activity decreases accordingly.

There was a significant positive correlation between soil extracellular enzyme activity and SOC, which supports the above findings (*p* < 0.05, Figure 8). This finding is consistent with that of Cheng et al. [31,44], who reported that soil enzyme activity is significantly correlated with SOC. There was a significant positive correlation between the activities of soil C, N, and P extracellular enzymes and microbial biomass (Figure 9). Soil microbial activity strongly depends on the accessibility of substrates [45]. Adequate nutrients and favorable soil moisture conditions promote a substantial increase in microbial growth [46], thereby promoting microbial anabolism [47]. In addition, soil pH is an important factor affecting soil enzyme activity [21]. Each enzyme has its own suitable pH range. Soil pH values that are too high or too low may lead to a decrease in enzyme activity. In this study, the activities of extracellular enzymes related to C, N, and P were significantly negatively correlated with pH (Figure 8). This is consistent with the results reported by Zhang et al. [21]. This may have occurred because vegetation planting increased litter and root exudates, acidified the soil, promoted microbial metabolism, and optimized enzyme activity [47].

### 4.2. Effects of Different Ecosystem Transition Models on Soil Extracellular Enzyme Stoichiometry

Soil extracellular enzyme stoichiometry can reflect strong correlations between microbial metabolic activity and soil resource availability [4,7]. In this study, compared with those in the CK treatment, the C:N_EEA_, C:P_EEA_, and N:P_EEA_ ratios of the soil extracellular enzymes all tended to decrease after ecosystem transition. This may be related to the contents of soil C and other nutrients. For example, the PO and AL treatments resulted in more significant decreases in the C:N_EEA_, C:P_EEA_, and N:P_EEA_ ratios (*p* < 0.05, Figure 3d–f). These findings indicate that C and other nutrients are key factors affecting soil enzyme activity and related stoichiometric changes. In different soil layers, as the soil depth increased, the C:N_EEA_, C:P_EEA_, and N:P_EEA_ ratios of the extracellular enzymes all tended to increase. According to Sinsabaugh et al. [7], the global average values of C:N_EEA_, C:P_EEA_, and N:P_EEA_ for extracellular enzymes are 1.41, 0.62, and 0.44, respectively. In this study, the enzyme C:N_EEA_ ratio in all the sample plots was less than the global average of 1.41, the C:P_EEA_ ratio was greater than the average of 0.62, and the N:P_EEA_ ratio was greater than the average of 0.44. These findings indicate that soil microbial metabolism in the study area is limited by Co-limitation of nitrogen and phosphorus. Wei et al. [6] reported that the soil C:N and C:P ratios in forest ecosystems strongly affect the C:N:P ratios of microorganisms and extracellular enzymes.

When soil microorganisms are limited by nutrients, microbial activity and metabolism are reduced, affecting nutrient cycling and the execution of ecological functions [48]. Previous studies have shown that on a global scale, the ratio ln(BG):ln(NAG + LAP):ln(Ap) of C, N, and P extracellular enzymes approaches 1:1:1 [7]. In this study, the slopes of ln(BG) and ln(NAG + LAP), ln(BG) and ln(AP) for each soil layer in the regression analysis all deviated from 1 (Figure 4a,b,d,e,g,h). The slopes of ln(NAG + LAP) and ln(AP) were close to 1 (Figure 4c,f,i), and the ln(BG):ln(NAG + LAP):ln(AP) ratio of soil extracellular enzymes was 1:1.83:1.54, deviating from the 1:1:1 ratio in the global ecosystem. These findings indicate that soil microbial nutrients are jointly limited by N and P and that soil nutrients affect soil extracellular enzyme activity by affecting soil microbial functions. Bi et al. [49] reported that the C:N:P ratio of enzymes in Pinus sylvestris plantations was 1:1.92:1.61. Huang et al. [38]. studied soils in mixed coniferous and broad-leaved forests and reported that the C:N:P ratio of enzymes was 1:0.92:1.68, which deviated from the global ratio of 1:1:1. When soil nutrient availability is low, microorganisms secrete more enzymes to meet nutrient demands [50]. This may occur due to the influence of vegetation and environmental factors on the biogeochemical cycle of soil elements, which results in the destruction of the dynamic balance in the enzymatic C:N:P ratio [25,49].

### 4.3. Nutrient Limitations of Soil Microorganisms Under Different Ecosystem Transition Models

The vector model was further used to characterize soil microbial nutrient limitations [12]. The changes in the vector length and vector angle of soil enzymes under different ecosystem transition modes indicated that microbial metabolism in surface soil was limited mainly by C and P and that microbial metabolism in the 10–20 cm and 20–40 cm layers was limited mainly by C and N (Figure 6a,b). This finding is consistent with the scatterplot results of soil enzyme stoichiometry (Figure 7a–c). There were also differences in microbial nutrient limitations among the different transition modes, with C limitations in the PO and AL treatments being significantly lower than those in CK. This may have occurred because the litter produced by the replanted vegetation decomposed, and the roots secreted more organic matter, which provided organic carbon for microorganisms and alleviated C limitations [38]. Similar results were also reported by Cui et al. [39], who revealed that the P limitation in the 0–10 cm soil layer was greatest in the CP treatment. With the exception of MM, P limitations on soil microbial metabolism increased in transition plots compared with CK. This may have occurred due to an increase in plant diversity and litter production with increased supply of C and N. This is consistent with Yang et al. [51], who reported that soil microbial metabolism was limited by P in a postfire regeneration model in northern coniferous forests in China.

The nutrient limitation in soil microorganisms changed from a P limitation in the 0–10 cm surface layer to an N limitation in the 10–20 cm and 20–40 cm soil layers. This shows that as the soil layer deepened, P limitation was alleviated, but N limitation intensified. On the one hand, it is possible that the area was located in a mountainous area with a large slope. Compared with flat areas, phosphorus loss is more likely to occur in sloped areas, leading to lower soil phosphorus availability [8]. On the other hand, this may have occurred because P mainly originates from the lithosphere and is difficult to obtain in large quantities from the atmosphere [52]. Phosphorus consumption by plants and adsorption by calcium and magnesium ions in alkaline soil reduce the availability of phosphorus during vegetation restoration [53], which results in P limitations in the surface layer. The 10–20 cm and 20–40 cm soil layers may be more limited by N because more phosphorus than nitrogen is released during the weathering of parent material in deep soil [33]. On the other hand, owing to the decomposition of litter by microorganisms, the nitrogen content of surface soil is greater than that of deep soil. Therefore, as soil depth increases, the amount of nitrogen available for plant uptake decreases, causing the soil to become N limited. This may occur due to changes in the soil N and P trends under the different vegetation restoration modes. This is consistent with the fact that microbial nutrient metabolism is jointly limited by N and P after vegetation restoration on the Loess Plateau [11,24].

In this study, a negative correlation between soil pH and the vector angle was observed. The vector angle increased with decreasing pH. This finding is consistent with the results of Zhang et al. [54], who revealed nutrient limitations in soil microorganisms in black locust forests. Soil pH is widely recognized as a key factor driving microbial community composition and activities [55], which may alter soil microbial nutrient limitations [56]. RDA revealed that changes in the activities and stoichiometry of soil extracellular enzymes can be attributed to changes in soil physical and chemical properties and microbial characteristics. In particular, the contribution rates of soil microbial properties were greater than those of soil physical and chemical properties (Figure 10a,b). The first two axes of soil microbial properties explained 85.72% of the total variation, which was much greater than the 59.42% explained by soil physical and chemical properties. In general, soil microorganisms are more sensitive to vegetation restoration than to changes in soil physical and chemical properties [21], mainly because soil microorganisms are directly involved in the secretion of enzymes [4]. These findings indicate that ecosystem transition in jujube forests affects the activity of extracellular enzymes and thereby affects soil nutrient cycling. Therefore, soil-related limitations in microbial metabolism during ecosystem transitions in jujube forests are the result of the comprehensive interactions among plants, soil, and microorganisms.

Overall, the ecosystem transition of jujube forests had a significant effect on the enhancement of soil nutrients. The replanting of vegetation favored the enhancement of nutrients, especially the replanting of trees (CP), which was more significant for each nutrient, followed by the replanting of herbs (AL), which was also more significant for the enhancement of SOC and N acquisition enzymes. Nutrient enhancement had a positive effect on microbiota and extracellular enzyme activities, which were positively correlated with microbial nutrient limitation. Since the region is mainly subject to the common limitation of N and P, the two treatments of CP and AL are more suitable for the ecosystem transition of red date palm forests in the Lüliang mountainous area relative to other modes. This provides a strong basis for the implementation of rational ecosystem transition in this region.

## 5. Conclusions

In this study, different modes of ecosystem transition significantly affected the carbon, nitrogen, and phosphorus contents of red jujube forest soils in the Lüliang Mountains, which in turn caused imbalances in C:N:P stoichiometry. The results showed that, compared with the CK treatment, the activities of soil C, N, and P extracellular enzymes significantly increased under revegetation with tree species. The log-transformed ratio of soil extracellular enzyme C:N:P in all plots in this study was 1:1.83:1.54, which deviated from the 1:1:1 ratio reported at the global ecosystem scale. Nutrient limitation in soil microorganisms changed from a P limitation to a N limitation as the soil layer deepened. As the soil layer deepened, C limitation increased, indicating that ecosystem transition in jujube forests is conducive to the alleviation of C and N limitations. Soil microbial properties better explained the effects of enzyme stoichiometry and nutrient limitation on microbial metabolism than soil physical and chemical properties did. Overall, the tree transplanting model improved soil nutrient levels more effectively than the herbaceous transplanting model. Therefore, in the management of various transformation models, it is crucial to consider the significant relationship between soil microorganisms and vegetation. Adopting the most suitable transformation model can enhance nutrient levels, maintain a harmonious balance of nutrient cycling within the soil ecosystem, and address the unique demands of limited nutrient supply.

## Data Availability

The original contributions presented in the study are included in the article, further inquiries can be directed to the corresponding author.
